# The impact of lung ultrasound on clinical-decision making across departments: a systematic review

**DOI:** 10.1186/s13089-021-00253-3

**Published:** 2022-01-10

**Authors:** Micah L. A. Heldeweg, Lian Vermue, Max Kant, Michelle Brouwer, Armand R. J. Girbes, Mark E. Haaksma, Leo M. A. Heunks, Amne Mousa, Jasper M. Smit, Thomas W. Smits, Frederique Paulus, Johannes C. F. Ket, Marcus J. Schultz, Pieter Roel Tuinman

**Affiliations:** 1 Department of Intensive Care Medicine, Amsterdam University Medical Centers, location VUmc, Postbox 7507, 1007MB Amsterdam, The Netherlands; 2 Amsterdam Leiden IC Echography (ALIFE), Amsterdam, The Netherlands; 3 Department of Intensive Care, Amsterdam University Medical Centers, location AMC, Amsterdam, The Netherlands; 4 Mahidol Oxford Tropical Medicine Research Unit (MORU), Mahidol University, Bangkok, Thailand; 5 Nuffield Department of Medicine, University of Oxford, Oxford, UK; 6 Medical Library, Amsterdam University Medical Centers, location VUmc, Amsterdam, The Netherlands

**Keywords:** Ultrasonography, Lung, Chest, Management, Clinical-decision making

## Abstract

**Background:**

Lung ultrasound has established itself as an accurate diagnostic tool in different clinical settings. However, its effects on clinical-decision making are insufficiently described. This systematic review aims to investigate the impact of lung ultrasound, exclusively or as part of an integrated thoracic ultrasound examination, on clinical-decision making in different departments, especially the emergency department (ED), intensive care unit (ICU), and general ward (GW).

**Methods:**

This systematic review was registered at PROSPERO (CRD42021242977). PubMed, EMBASE, and Web of Science were searched for original studies reporting changes in clinical-decision making (e.g. diagnosis, management, or therapy) after using lung ultrasound. Inclusion criteria were a recorded change of management (in percentage of cases) and with a clinical presentation to the ED, ICU, or GW. Studies were excluded if examinations were beyond the scope of thoracic ultrasound or to guide procedures. Mean changes with range (%) in clinical-decision making were reported. Methodological data on lung ultrasound were also collected. Study quality was scored using the Newcastle–Ottawa scale.

**Results:**

A total of 13 studies were included: five studies on the ED (546 patients), five studies on the ICU (504 patients), two studies on the GW (1150 patients), and one study across all three wards (41 patients). Lung ultrasound changed the diagnosis in mean 33% (15–44%) and 44% (34–58%) of patients in the ED and ICU, respectively. Lung ultrasound changed the management in mean 48% (20–80%), 42% (30–68%) and 48% (48–48%) of patients in the ED, in the ICU and in the GW, respectively. Changes in management were non-invasive in 92% and 51% of patients in the ED and ICU, respectively. Lung ultrasound methodology was heterogeneous across studies. Risk of bias was moderate to high in all studies.

**Conclusions:**

Lung ultrasound, exclusively or as a part of thoracic ultrasound, has substantial impact on clinical-decision making by changing diagnosis and management in the EDs, ICUs, and GWs. The current evidence level and methodological heterogeneity underline the necessity for well-designed trials and standardization of methodology.

**Supplementary Information:**

The online version contains supplementary material available at 10.1186/s13089-021-00253-3.

## Background

Lung ultrasound is a rapidly growing point-of-care diagnostic and monitoring modality in emergency departments (EDs), intensive care units (ICUs), and general wards (GWs). It is an increasingly common addition to standard physical examination and has been included in many specialist training programs [1–3]. Lung ultrasound’s advantages are that it is non-invasive, easy to learn, and accurate in discriminating pulmonary pathology [4–6]. Moreover, integrating lung ultrasound with bedside cardiac and caval ultrasound expands its utility to comprehensively assess cardiopulmonary status [7]. Clinical use of lung ultrasound may therefore allow quicker arrival at correct diagnosis and management leading to improved patient outcomes.

Lung ultrasound’s prompt emergence is also accompanied with several knowledge gaps. Previous literature has addressed issues such as methodological heterogeneity and reproducibility [8, 9]. However, whether the implementation of lung ultrasound affects clinical-decision making remains largely unaddressed.

This systematic review investigates how often use of lung ultrasound, exclusively or as a part of thoracic ultrasound, leads to changes in clinical-decision making across departments, e.g. ED, ICU and GW.

## Methods

This systematic review was registered at the International Prospective Register of Systematic Reviews (CRD42021242977). The Preferred Reporting Items for Systematic Reviews and Meta-analysis guidelines were followed to safeguard transparent and complete reporting of our review.

### Search strategy

A comprehensive search of available literature on impact of lung ultrasound on clinical-decision making was built with the help of a medical librarian. Medical subject headings, keywords, and synonyms pertaining to lung, ultrasound, decision making, and patient management were used to search PubMed (Medline) up to March 19th 2021, as well as Embase and Web of Science up to April 6th 2021 (Additional file 1).

### Study selection and inclusion

Inclusion criteria for studies were: (i) Original research in the English language reporting on changes in clinical-decision making following lung ultrasound examination exclusively or as a part of thoracic ultrasound. Clinical-decision making was defined as either a clinician’s diagnosis, management, or therapy. At least a recorded change of management, the overarching term encompassing changes of therapy, changes of level of care, disposition, or consultations was required for inclusion; (ii) Patients in the ED, ICU (including medium care unit), or GW; (iii) General clinical presentations including hemodynamic or respiratory instability.

Exclusion criteria were: (i) Examinations beyond the scope of thoracic ultrasound (e.g. transcranial or abdominal); (ii) Ultrasound used solely for guiding procedures; (iii) Outpatient, prehospital, or rural patient presentations; (iv) Specific clinical presentations (e.g. only patients with pulmonary embolism). Title and abstract screening was conducted by two investigators (LV and MK) and conflicts were resolved with help of a third investigator (MLAH).

### Data extraction

Two investigators (MLAH and LV) extracted the following data from each study: (i) population; (ii) diagnostic and/or monitoring protocol; (iii) reported clinical-decision making change(s). Changes in therapy were further subdivided into non-invasive (pharmacological, fluids, ventilator settings, physiotherapy) and invasive (surgical procedure, start or stop mechanical ventilation, invasive diagnostic, or dialysis); (iv) methodological aspects (probe, lung ultrasound scoring system, training, interrater agreement).

### Data synthesis

Studies were categorized according to setting (ED, ICU, or GW). Depending on the availability of data, the impact on clinical-decision was shown as a number and percentage (%) of change in diagnosis, change in management, and change in therapy. Meta-analysis was considered inappropriate due to anticipated heterogeneity and lack of inferential statistics. As data is restricted to a limited number of studies, total changes across studies were presented as mean with reported range to facilitate interpretation. Other reported metrics on clinical-decision making were textually presented when appropriate. Although multiple changes of management can occur in one examination, when possible, the results were presented as relative number of examinations leading to changes to enable the calculation of a percentage.

Lung ultrasound’s major paradigm shift occurred after a landmark April 2008 study [10]. Studies before this date had significantly different lung ultrasound methodologies. These studies primarily relied on limited lung ultrasound protocols, stricter patient selection, and external sonographers further removed from clinical decisions. These studies were not included in the aggregate, but discussed separately.

### Quality assessment

The Newcastle–Ottawa scale for cohort studies was used to assess risk of bias. This quality assessment tool classifies an observational study’s risk of bias with 0–9 stars, which was further subdivided as high (0–3 stars), moderate (4–6 stars), or low (7–9 stars) quality [11].

## Results

**Fig. 1:**
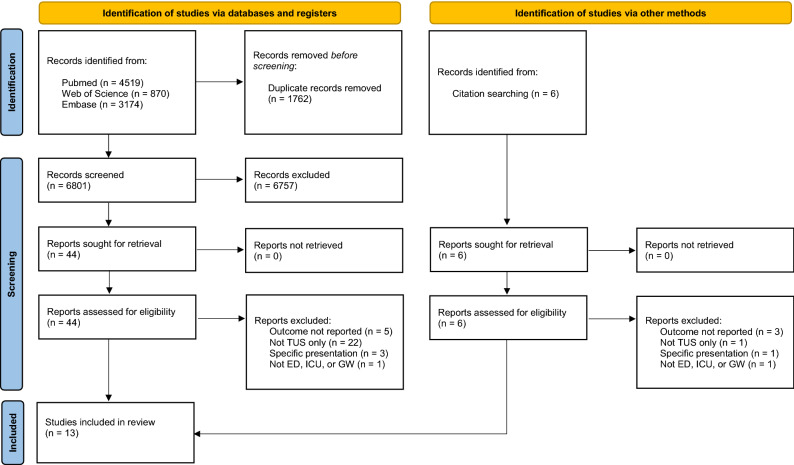
PRISMA flow diagram. TUS thoracic ultrasound; ED emergency department; ICU intensive care unit; GW general ward

A total of 8563 records were identified through the search in PubMed (53%), Web of Science (10%), and Embase (37%). An additional six records were identified by screening reference lists. After removal of duplicates and screening using inclusion and exclusion criteria, 50 records were read in full. Ultimately, 13 studies with a total of 2142 patients were included for data extraction (Fig. 1).

All 13 studies had an observational design. Five studies were performed in the ED, five at the ICU, two at the GW, and one at all three different departments. In the 10 studies performed after 2008 lung ultrasound was effectuated by the bedside clinician or investigators. In the three studies performed before April 2008, lung ultrasound was performed by independent radiologists or technicians. Five studies only investigated lung ultrasound, four included cardiac ultrasound, one included caval ultrasound, and one combined all thoracic ultrasound modalities.

### Effects of ultrasound on clinical-decision making in ED patients

Outcomes of the five studies performed in the ED are presented in Table 1. Four studies investigated patients with dyspnea [12–15]. One study, performed in 2001, investigated patients with “acute chest symptoms” [16].

**Table 1:** Effect of ultrasound on clinical-decision making reported by ED studies

Study	Year	Patients (n), symptom	Ultrasound	Diagnosis change	Management change	Therapy change	Type of therapy changes
*House*	2020	280, dyspnea	Lung	124 (44.3%)	150 (53.6%)	125 (44.6%)	Invasive 9/125 n-Invasive 116/125
*Shah*	2016	117, dyspnea	Lung + cardiac	18 (15.4%)	23 (19.6%)	23 (19.6%)	Invasive 1/23 n-Invasive 22/23
*Russell*	2015	99, dyspnea	Lung + cardiac + caval	17 (17%)	47 (47%)	42 (42%)	Invasive 2/42 n-Invasive 40/42
*Goffi*	2013	50, dyspnea	Lung	22 (44%)	40 (80%)	35 (70%)	Invasive 6/35 n-Invasive 29/35
Total		546		181 (33.2%)	260 (47.6%)	225 (41.2%)	Invasive 18/225 n-invasive 207/225
*Yuan*	2001	78 acute chest symptoms	Lung + cardiac	52 (66.7%)	35 (44.9%)	34 (43.6%)	Invasive 17/34 n-Invasive 17/34

TOTAL referred to the compilation of studies published after April 2008; ED emergency department

Changes in diagnosis, management, and therapy occurred in 33.2% (15.4–44%), 47.6% (19.6–80%), and 41.2% (19.6–70%), respectively. Of therapy changes 92% were non-invasive. Two studies included patients < 18 years. One reported that changes in clinical impression (13.7%) and changes in diagnosis (3.4%) were less frequent in the pediatric population compared to adults (24.8% of total population, mean age 3.4 years), whilst the other study did not specify any age-related characteristics [12, 13].

### Effects of ultrasound on clinical-decision making in ICU patients

Outcomes of five studies performed in the ICU are presented in Table 2. Two studies evaluated patients with respiratory failure and two studies evaluated mechanically ventilated patients [17–20]. One ICU study, performed in 2008, evaluated the impact of “chest sonography” on all ICU patients during five months of on-demand chest radiography and five months of daily routine chest radiography [21].

**Table 2:** Effect of ultrasound on clinical-decision making reported by ICU studies

Study	Year	Patients (n), symptom	Ultrasound	Diagnosis change	Management change	Type of changes
Barman	2020	108, respiratory failure	Lung + cardiac	40 (37%)	39 (36%)	Invasive 44/69 n-Invasive 25/69
Haji	2018	93, unspecified	Lung + cardiac	53 (58%)	60 (68%)	Invasive 2/60 n-Invasive 58/60
Wallbridge	2017	50, respiratory failure	Lung + caval	17 (34%)	15 (30%)	Invasive 1/15 n-Invasive 14/15
Xirouchaki	2014	253, MV adults	Lung	N/A	119 (47%)	Invasive 81/119 n-Invasive 38/119
Total		504		110 (43.8%)	233 (42.2%)	Invasive 128/263 n-Invasive 135/263
Kröner	2008	36 adults	Lung	16 of 43 (37.2%)	18 of 48 (38%)	N/A

MV mechanically ventilated; N/A not available; TOTAL referred to the compilation of studies published after April 2008; *ICU* intensive care unit

Changes in diagnosis and management occurred in 43.8% (34—58%) and 42.2% (30–68%), respectively. Of therapy changes 51% were non-invasive. Rather than reporting changes in diagnosis, one study reported the net reclassification index (85.6%) as a metric for the impact of lung ultrasound on decision making [20]. According to one of the studies, patients who had multiple initial clinical diagnoses were more likely to have a change in management following ultrasound scanning (8/16 patients, compared to 7/34 patients with a single diagnosis, *p* = 0.034) [19]. None of the ICU studies differentiated between management or therapy changes.

### Effects of ultrasound on clinical-decision making in GW patients

Two studies were performed on the GW with a mean change of management in 47.7% of examinations. Both studies did not report change in diagnosis or types of changes. On the Internal Medicine Ward, lung ultrasound led to a change in management in 25 out of 52 (48.0%) examinations [22]. Pulmonologists used lung ultrasound across different wards to influence clinical-decision making, including treatment, in 548 (47.7%) out of 1150 examinations [23].

A study from 1992, showed that chest sonographers’ examinations on ED (*n* = 6), ICU (*n* = 19), and GW (*n* = 16) patients, assisted diagnosis in 27 (66%) patients and changed management in 25 (60.9%) of 41 patients [24].

### Lung ultrasound methodology

Table 3 shows a large variance in lung ultrasound methodology across included studies. The most frequently used lung ultrasound protocol was 8-zone with a convex probe oriented perpendicular to the ribs.

**Table 3:** Lung ultrasound methodology of included studies

Study	Zones	Orientation	B-line appraisal	Probe	Examiner	Interrater agreement
ED						
House 2020	10	Perpendicular	≥ 2 positive regions with ≥ 3 B-lines	Convex	Clinician + trained	Experts: 0.9 Clinician: 0.8
Shah 2016	18	Perpendicular	≥ 2 positive regions with ≥ 3 B-lines	Phased	Clinician + trained	LVEF κ:0.98
Russell 2015	8	Perpendicular	≥ 2 positive regions with ≥ 4 B-lines	Convex	Investigator + trained	Investigators κ: 0.82
Goffi 2013	8	Perpendicular	≥ 2 positive regions with ≥ 3 B-lines	Convex	Investigator	N/A
Yuan 2001	N/A	N/A	N/A	Linear + convex + phased	Technician + trained	N/A
ICU						
Barman 2020	8	Parallel	≥ 2 positive regions with ≥ 3 B-lines	Linear + convex	Investigator	N/A
Haji 2018	12	Perpendicular	≥ 2 positive regions with ≥ 3 B-lines	N/A	Investigator + experience	κ:0.69
Wallbridge 2017	N/A	Parallel	≥ 2 zones with B-lines: diffuse	Convex + linear	Investigator + certified	N/A
Xirouchaki 2013	12	Perpendicular	> 1 B-line in zone	Convex	Investigator + experience	N/A
Kröner 2008	N/A	N/A	N/A	N/A	Technician	N/A
GW						
Mozzini 2016	28/8/2	Perpendicular	≥ 2 positive regions with ≥ 3 B-lines	Linear + convex + phased	Clinicians + trained	Various
Sferrazza papa 2016	8	Perpendicular	≥ 2 positive regions with ≥ 3 B-lines	Convex + linear	Clinicians + trained	N/A
Yu 1992	N/A	N/A	N/A	Convex + linear + phased	Technician	N/A

κ kappa degree of agreement; N/A not available. The probes were grouped in major probe categories (e.g. phased, convex, linear) although their specific frequency range varied. The examiner was described as investigator, technician, or clinician. Training of examiners were grouped into experienced, trained and certified although the respective definition of the former varied substantially

### Quality assessment

Table 4 shows the quality assessment. Four studies exhibit a high risk of bias, and nine studies a moderate risk of bias (4–6). None of the studies offer comparable control arms for clinical-decision making.

**Table 4:** Quality assessment of studies for this systematic review’s outcome of interest using the Newcastle–Ottawa scale for cohort studies

	Selection				Comparability	Outcomes			Total
	Representativeness	Selection of non-exposed	Ascertainment of exposure	Outcome not present at start of study	Comparability of cohorts on the basis of the design of analysis	Assessment of outcome	Sufficient follow up time	Adequacy of follow up of cohorts	
House 2020	★	☆	★	★	☆☆	★	★	★	6/9
Shah 2016	★	☆	☆	★	☆☆	★	★	★	5/9
Russel 2015	★	☆	★	★	☆☆	★	★	★	6/9
Goffi 2013	★	☆	☆	★	☆☆	☆	★	★	4/9
Yuan 2001	★	☆	☆	★	☆☆	☆	★	★	4/9
Barman 2020	★	☆	☆	☆	☆☆	☆	★	★	3/9
Haji 2018	★	☆	★	★	☆☆	★	★	★	6/9
Wallbridge 2017	★	☆	☆	★	☆☆	☆	★	★	4/9
Xirouchaki 2013	★	☆	☆	☆	☆☆	☆	★	★	3/9
Kröner 2008	★	☆	☆	★	☆☆	★	★	★	5/9
Mozzini 2016	★	☆	☆	☆	☆☆	☆	★	★	3/9
Sferrazza 2016	★	☆	★	★	☆☆	☆	★	★	5/9
Yu 1992	☆	☆	☆	★	☆☆	☆	★	★	3/9

Empty stars reflects lack of sufficient quality on the respective domains. Full starts reflect sufficient quality on respective domains where total represents high (0–3 stars), moderate (4–6 stars), or low (7–9 stars) risk of bias

## Discussion

The main findings of this systematic review are: (i) Lung ultrasound resulted in a large proportion of diagnosis changes in the ED and ICU (34% and 44% of examinations, respectively); (ii) Lung ultrasound resulted in substantial management changes in the ED, ICU, and GW (48%, 42%, and 48% of examinations, respectively); (iii) On the ED and ICU therapy changes were most frequently non-invasive (92% and 51% respectively); (iv) Lung ultrasound methodology was heterogeneous across studies; v. Moderate to high risk of bias was present across all studies.

This study shows that bedside lung ultrasound is frequently a decisive tool in different clinical settings. This is an important finding: changes to physician behavior and subsequent modification of patient care might result in improvement in patient-centered outcomes. Moreover, even when no changes are effectuated, the confirmation of clinical impression could prevent uptake of further, costly or more invasive diagnostic and monitoring modalities. Additionally, the outcomes of the included studies were absolute (change versus no change), but ultrasound may also induce modification of prior likelihood. Resulting elimination of uncertainty may prevent delays of indicated care and, in some cases, patient harm [25, 26].

This study found that the majority of ultrasound-induced changes were classified as non-invasive as opposed to invasive. This classification is limited in evaluating true effects of changes, both in diagnosis and management, on patient-centered outcomes. For example, a non-invasive change may be to abstain from increasing furosemide dose, but another may be to start rapid fluid resuscitation. At the same time, small changes in management should not be underestimated, as, for example, optimization of volume status may result in faster liberation from mechanical ventilation [27].

Consistent with previous literature, the majority of studies integrated lung ultrasound with other thoracic ultrasound modalities. This is a reasonable approach as pathologies encountered upon thoracic ultrasound are often (patho)physiologically linked. Findings on lung ultrasound may support, modify, or moderate cardiac and caval ultrasound’s findings and vice versa. Moreover, previous research also showed that an integrated thoracic ultrasound approach performs better than its individual components [28]. Other studies have expanded bedside ultrasound beyond thorax to include transcranial and abdominal ultrasound and found even higher impact on clinical management (60% and 69%, respectively) [29, 30]. Evidently, point-of-care ultrasound modalities, combined or separately, have a substantial impact on clinical-decision making.

The current study examines the use of lung ultrasound in ED, ICU, and GW; three hospital departments where point-of-care ultrasound is very relevant. Results across these hospital settings were similar. Interestingly, the use of point-of-care ultrasound has expanded beyond hospital medicine. One study showed that the introduction of point-of-care ultrasound in general practice alters the diagnostic process and results in changes of diagnosis and management in half of patients [31]. Similarly, prehospital (and rural) studies employing a wide variety of POCUS examinations found a significant benefit that can dramatically alter disposition and treatment (50% of patients) and correlated well with in-hospital diagnostic results [32, 33].

The methodology and quality assessment tables highlight weaknesses in current lung ultrasound research. Methodological inconsistencies are frequent amongst lung ultrasound investigations, may impact findings, and limit clinical reproducibility or generalizability [8, 9, 34]. The lack of comparator for any of the studies may be intrinsic to the selected outcome, but emphasizes the need for controlled and well-designed studies to study the effect of lung ultrasound beyond clinician behavior: patient-centered and hospital level outcomes. Even excellent diagnostic tools do not necessarily lead to improved patient-centered outcomes [35]. Similarly, wrongful interpretation of ultrasound accompanied by unwarranted change of management may have undesired effects. Currently, several trials are either underway or recently published that may potentially provide higher quality evidence [36–38].

This is the first study that systematically and exhaustively, including a total of 13 studies and 2142 patients, describes the impact of lung ultrasound on clinical-decision making. The search strategy was extensive (including three databases) to enhance identification of relevant studies. Strict methodology was used, including inclusion and exclusion criteria to increase homogeneity across studies, a recurring issue in ultrasound literature.

A limitation to the current study is that the outcome of interest, physician behavior, is not necessarily associated with improvement of patient-centered outcomes. Assessment of the latter would require randomized or blinded studies to avoid confounding factors. Moreover, ultrasound-driven intended changes in physician behavior may not necessarily be executed. Feasibility studies are required to assess actual management effectuation. Furthermore, assessment of publication bias, e.g. by a funnel-plot, was not done. Lastly, it is possible that not all studies were identified due to the requirement of English language.

## Conclusions

Lung ultrasound, exclusively or as a part of thoracic ultrasound, has a major impact on clinical-decision making by changing diagnosis management, and therapy in different clinical settings. However, the current evidence level and methodological heterogeneity underlines the invariable necessity for well-designed trials and standardization of ultrasound methodology.

## Supplementary Information

**Additional file 1.** a: First PubMed search 3575. b: Thorax added 944 to PubMed search. c: Embase search. d: Thorax added to Embase search. e: Web of Science search. f: Thorax added to Web of Science search.

## Data Availability

The datasets used and/or analyzed during the current study are available from the corresponding author on reasonable request.

## Abbreviations

ED: Emergency Department
GW: General ward
ICU: Intensive Care Unit
