# Supplementary material for: The impact of lung ultrasound on clinical-decision making across departments: a systematic review
Source: Ultrasound J. 2022 Jan 10;14:5. doi: 10.1186/s13089-021-00253-3 (PMC8748548; doi:10.1186/s13089-021-00253-3)
Supplement: Supplementary file 1 — Additional file 1. a: First PubMed search 3575. b: Thorax added 944 to PubMed search. c: Embase search. d: Thorax added to Embase search. e: Web of Science search. f: Thorax added to Web of Science search. [file 13089_2021_253_MOESM1_ESM.docx]

**Additional file 1a: First PubMed search 3575**

| **#5** | **#4 NOT (animals[mh] NOT humans[mh])** | [**3,575**](https://pubmed.ncbi.nlm.nih.gov/?term=%234+NOT+(animals%5Bmh%5D+NOT+humans%5Bmh%5D)&sort=date&size=200&ac=no) |
| --- | --- | --- |
| **#4** | **#1 AND #2 AND #3** | [**3,759**](https://pubmed.ncbi.nlm.nih.gov/?term=%231+AND+%232+AND+%233&sort=date&size=200&ac=no) |
| **#3** | **"Clinical Decision-Making"[Mesh] OR "Decision Making"[Mesh] OR (("manag*"[tiab] OR "therap*"[tiab] OR "interven*"[tiab]) AND ("chang*"[tiab] OR "impact*"[tiab] OR "decision*"[tiab])) OR "decision making"[tiab] OR "clinical reasoning*"[tiab] OR "clinical judgement*"[tiab] OR "clinical judgment*"[tiab]** | [**1,297,815**](https://pubmed.ncbi.nlm.nih.gov/?term=%22Clinical+Decision-Making%22%5BMesh%5D+OR+%22Decision+Making%22%5BMesh%5D+OR+((%22manag*%22%5Btiab%5D+OR+%22therap*%22%5Btiab%5D+OR+%22interven*%22%5Btiab%5D)+AND+(%22chang*%22%5Btiab%5D+OR+%22impact*%22%5Btiab%5D+OR+%22decision*%22%5Btiab%5D))+OR+%22decision+making%22%5Btiab%5D+OR+%22clinical+reasoning*%22%5Btiab%5D+OR+%22clinical+judgement*%22%5Btiab%5D+OR+%22clinical+judgment*%22%5Btiab%5D&sort=date&size=200) |
| **#2** | **"Ultrasonography"[Mesh] OR "ultraso*"[tiab] OR "echogra*"[tiab] OR "echotomogra*"[tiab] OR "echosound*"[tiab] OR "sonogra*"[tiab] OR "echo"[tiab]** | [**703,570**](https://pubmed.ncbi.nlm.nih.gov/?term=%22Ultrasonography%22%5BMesh%5D+OR+%22ultraso*%22%5Btiab%5D+OR+%22echogra*%22%5Btiab%5D+OR+%22echotomogra*%22%5Btiab%5D+OR+%22echosound*%22%5Btiab%5D+OR+%22sonogra*%22%5Btiab%5D+OR+%22echo%22%5Btiab%5D&sort=date&size=200) |
| **#1** | **"Lung"[Mesh] OR "Lung Diseases"[Mesh] OR "lung"[tiab] OR "lungs"[tiab] OR "cardiopulmonar*"[tiab] OR "pulmo*"[tiab] OR "pleuropulmonar*"[tiab] OR "bronchopulmonar*"[tiab] OR "pleura*"[tiab] OR "non cardiogen*"[tiab] OR "dyspnea*"[tiab] OR "dyspnoea*"[tiab]** | [**1,687,823**](https://pubmed.ncbi.nlm.nih.gov/?term=%22Lung%22%5BMesh%5D+OR+%22Lung+Diseases%22%5BMesh%5D+OR+%22lung%22%5Btiab%5D+OR+%22lungs%22%5Btiab%5D+OR+%22cardiopulmonar*%22%5Btiab%5D+OR+%22pulmo*%22%5Btiab%5D+OR+%22pleuropulmonar*%22%5Btiab%5D+OR+%22bronchopulmonar*%22%5Btiab%5D+OR+%22pleura*%22%5Btiab%5D+OR+%22non+cardiogen*%22%5Btiab%5D+OR+%22dyspnea*%22%5Btiab%5D+OR+%22dyspnoea*%22%5Btiab%5D&sort=date&size=200) |

**Additional file 1b: Thorax added 944 to PubMed search**

| **#8** | **#7 NOT (animals[mh] NOT humans[mh])** | [**944**](https://pubmed.ncbi.nlm.nih.gov/?term=%237+NOT+(animals%5Bmh%5D+NOT+humans%5Bmh%5D)&sort=date&ac=no) |
| --- | --- | --- |
| **#7** | **#5 NOT #6** | [**1,066**](https://pubmed.ncbi.nlm.nih.gov/?term=%235+NOT+%236&sort=date) |
| **#6** | **#1 AND #2 AND #4** | [**11,310**](https://pubmed.ncbi.nlm.nih.gov/?term=%231+AND+%232+AND+%234&sort=date&ac=no) |
| **#5** | **#1 AND #2 AND #3** | [**1,790**](https://pubmed.ncbi.nlm.nih.gov/?term=%231+AND+%232+AND+%233&sort=date&ac=no) |
| **#4** | **"Lung"[Mesh] OR "Lung Diseases"[Mesh] OR "lung"[tiab] OR "lungs"[tiab] OR "cardiopulmonar*"[tiab] OR "pulmo*"[tiab] OR "pleuropulmonar*"[tiab] OR "bronchopulmonar*"[tiab] OR "pleura*"[tiab] OR "non cardiogen*"[tiab] OR "dyspnea*"[tiab] OR "dyspnoea*"[tiab]** | [**1,686,690**](https://pubmed.ncbi.nlm.nih.gov/?term=%22Lung%22%5BMesh%5D+OR+%22Lung+Diseases%22%5BMesh%5D+OR+%22lung%22%5Btiab%5D+OR+%22lungs%22%5Btiab%5D+OR+%22cardiopulmonar*%22%5Btiab%5D+OR+%22pulmo*%22%5Btiab%5D+OR+%22pleuropulmonar*%22%5Btiab%5D+OR+%22bronchopulmonar*%22%5Btiab%5D+OR+%22pleura*%22%5Btiab%5D+OR+%22non+cardiogen*%22%5Btiab%5D+OR+%22dyspnea*%22%5Btiab%5D+OR+%22dyspnoea*%22%5Btiab%5D&sort=date&ac=no) |
| **#3** | **"Clinical Decision-Making"[Mesh] OR "Decision Making"[Mesh] OR (("manag*"[tiab] OR "therap*"[tiab] OR "interven*"[tiab]) AND ("chang*"[tiab] OR "impact*"[tiab] OR "decision*"[tiab])) OR "decision making"[tiab] OR "clinical reasoning*"[tiab] OR "clinical judgement*"[tiab] OR "clinical judgment*"[tiab]** | [**1,297,092**](https://pubmed.ncbi.nlm.nih.gov/?term=%22Clinical+Decision-Making%22%5BMesh%5D+OR+%22Decision+Making%22%5BMesh%5D+OR+((%22manag*%22%5Btiab%5D+OR+%22therap*%22%5Btiab%5D+OR+%22interven*%22%5Btiab%5D)+AND+(%22chang*%22%5Btiab%5D+OR+%22impact*%22%5Btiab%5D+OR+%22decision*%22%5Btiab%5D))+OR+%22decision+making%22%5Btiab%5D+OR+%22clinical+reasoning*%22%5Btiab%5D+OR+%22clinical+judgement*%22%5Btiab%5D+OR+%22clinical+judgment*%22%5Btiab%5D&sort=date&ac=no) |
| **#2** | **"Ultrasonography"[Mesh] OR "ultraso*"[tiab] OR "echogra*"[tiab] OR "echotomogra*"[tiab] OR "echosound*"[tiab] OR "sonogra*"[tiab] OR "echo"[tiab]** | [**703,369**](https://pubmed.ncbi.nlm.nih.gov/?term=%22Ultrasonography%22%5BMesh%5D+OR+%22ultraso*%22%5Btiab%5D+OR+%22echogra*%22%5Btiab%5D+OR+%22echotomogra*%22%5Btiab%5D+OR+%22echosound*%22%5Btiab%5D+OR+%22sonogra*%22%5Btiab%5D+OR+%22echo%22%5Btiab%5D&sort=date&ac=no) |
| **#1** | **"Thorax"[Mesh] OR "thora*"[tiab] OR "chest*"[tiab]** | [**396,737**](https://pubmed.ncbi.nlm.nih.gov/?term=%22Thorax%22%5BMesh%5D+OR+%22thora*%22%5Btiab%5D+OR+%22chest*%22%5Btiab%5D&sort=date&ac=no) |

**Additional file 1c: Embase search**

| **#12** | #11 NOT ('conference abstract'/it OR 'conference paper'/it OR 'conference review'/it) | **2629** |
| --- | --- | --- |
| **#11** | #10 NOT ([animals]/lim NOT [humans]/lim) | **5141** |
| **#10** | #1 AND #2 AND #9 | **5197** |
| **#9** | #7 OR #8 | **613476** |
| **#8** | ('manag*' OR 'therap*' OR 'interven*') NEAR/3 ('chang*' OR 'impact*' OR 'decision*') | **159526** |
| **#7** | 'decision making'/exp OR 'change management'/exp OR 'clinical reasoning'/exp OR 'decision making':ti,ab,kw OR 'clinical reasoning*':ti,ab,kw OR 'clinical judgement*':ti,ab,kw OR 'clinical judgment*':ti,ab,kw | **476327** |
| **#2** | 'echography'/exp OR 'ultraso*':ti,ab,kw OR 'echogra*':ti,ab,kw OR 'echotomogra*':ti,ab,kw OR 'echosound*':ti,ab,kw OR 'sonogra*':ti,ab,kw OR 'echo':ti,ab,kw | **1224788** |
| **#1** | 'lung'/exp OR 'lung disease'/exp OR 'lung':ti,ab,kw OR 'lungs':ti,ab,kw OR 'cardiopulmonar*':ti,ab,kw OR 'pulmo*':ti,ab,kw OR 'pleuropulmonar*':ti,ab,kw OR 'bronchopulmonar*':ti,ab,kw OR 'pleura*':ti,ab,kw OR 'non cardiogen*':ti,ab,kw OR 'dyspnea*':ti,ab,kw OR 'dyspnoea*':ti,ab,kw | **2394268** |

**Additional file 1d: Thorax added to Embase search**

| **No.** | **Query** | **Results** |
| --- | --- | --- |
| **#9** | #8 NOT [2629 PUIs] | **545** |
| **#8** | #7 NOT ('conference abstract'/it OR 'conference paper'/it OR 'conference review'/it) | **1106** |
| **#7** | #6 NOT ([animals]/lim NOT [humans]/lim) | **2876** |
| **#6** | #1 AND #2 AND #5 | **2922** |
| **#5** | #3 OR #4 | **614038** |
| **#4** | ('manag*' OR 'therap*' OR 'interven*') NEAR/3 ('chang*' OR 'impact*' OR 'decision*') | **159692** |
| **#3** | 'decision making'/exp OR 'change management'/exp OR 'clinical reasoning'/exp OR 'decision making':ti,ab,kw OR 'clinical reasoning*':ti,ab,kw OR 'clinical judgement*':ti,ab,kw OR 'clinical judgment*':ti,ab,kw | **476738** |
| **#2** | 'echography'/exp OR 'ultraso*':ti,ab,kw OR 'echogra*':ti,ab,kw OR 'echotomogra*':ti,ab,kw OR 'echosound*':ti,ab,kw OR 'sonogra*':ti,ab,kw OR 'echo':ti,ab,kw | **1225877** |
| **#1** | 'thorax'/exp OR 'thora*':ti,ab,kw OR 'chest*':ti,ab,kw | **655774** |

**Additional file 1e: Web of Science search**

| **Set** | **Results** | **Query** |
| --- | --- | --- |
| **#6** | **290** | #5 NOT [2994 PMIDs] |
| **#5** | **297** | #4 NOT [2998 PMIDs] |
| **#4** | [**669**](http://apps.webofknowledge.com.vu-nl.idm.oclc.org/summary.do?product=WOS&doc=1&qid=4&SID=E42fSbJNiiLst3LF1Xs&search_mode=CombineSearches&update_back2search_link_param=yes) | #3 AND #2 AND #1 |
| **#3** | [**501,677**](http://apps.webofknowledge.com.vu-nl.idm.oclc.org/summary.do?product=WOS&doc=1&qid=3&SID=E42fSbJNiiLst3LF1Xs&search_mode=GeneralSearch&update_back2search_link_param=yes) | **TOPIC:** ((("manag*" OR "therap*" OR "interven*") NEAR/3 ("chang*" OR "impact*" OR "decision*") ) OR "decision making" OR "clinical reasoning*" OR "clinical judgement*" OR "clinical judgment*") |
| **#2** | [**638,133**](http://apps.webofknowledge.com.vu-nl.idm.oclc.org/summary.do?product=WOS&doc=1&qid=2&SID=E42fSbJNiiLst3LF1Xs&search_mode=GeneralSearch&update_back2search_link_param=yes) | **TOPIC:** ("ultraso*" OR "echogra*" OR "echotomogra*" OR "echosound*" OR "sonogra*" OR "echo") |
| **#1** | [**1,351,658**](http://apps.webofknowledge.com.vu-nl.idm.oclc.org/summary.do?product=WOS&doc=1&qid=1&SID=E42fSbJNiiLst3LF1Xs&search_mode=GeneralSearch&update_back2search_link_param=yes) | **TOPIC:** ("lung" OR "lungs" OR "cardiopulmonar*" OR "pulmo*" OR "pleuropulmonar*" OR "bronchopulmonar*" OR "pleura*" OR "non cardiogen*" OR "dyspnea*" OR "dyspnoea*") |

**Additional file 1f: Thorax added to Web of Science search**

| **Set** | **Results** | **Query** |
| --- | --- | --- |
| **#7** | **201** | #4 NOT #6 |
| **#6** | **671** | #5 AND #3 AND #2 |
| **#5** | **1,353,408** | TS=("lung" OR "lungs" OR "cardiopulmonar*" OR "pulmo*" OR "pleuropulmonar*" OR "bronchopulmonar*" OR "pleura*" OR "non cardiogen*" OR "dyspnea*" OR "dyspnoea*") |
| **#4** | **413** | #3 AND #2 AND #1 |
| **#3** | **502,812** | TOPIC: ((("manag*" OR "therap*" OR "interven*") NEAR/3 ("chang*" OR "impact*" OR "decision*") ) OR "decision making" OR "clinical reasoning*" OR "clinical judgement*" OR "clinical judgment*") |
| **#2** | **638,912** | TOPIC: ("ultraso*" OR "echogra*" OR "echotomogra*" OR "echosound*" OR "sonogra*" OR "echo") |
| **#1** | **353,978** | TOPIC: ("thora*" OR "chest*") |
